# Prevalence of loneliness and social isolation among individuals with mild cognitive impairment or dementia: systematic review and meta-analysis

**DOI:** 10.1192/bjo.2024.865

**Published:** 2025-03-11

**Authors:** André Hajek, Hans-Helmut König

**Affiliations:** Department of Health Economics and Health Services Research, University Medical Center Hamburg-Eppendorf, Hamburg Center for Health Economics, Hamburg, Germany

**Keywords:** Dementia, loneliness, mild cognitive impairment, social exclusion, social isolation

## Abstract

**Background:**

A systematic review/meta-analysis synthesising the existing evidence regarding the prevalence of loneliness and social isolation among individuals with mild cognitive impairment (MCI) or dementia is lacking.

**Aims:**

A systematic review and meta-analysis was conducted to investigate the prevalence and factors associated with loneliness and social isolation among individuals with MCI or dementia.

**Method:**

A search was conducted in five established electronic databases. Observational studies reporting prevalence and, where available, factors associated with loneliness/isolation among individuals with MCI and individuals with dementia, were included. Important characteristics of the studies were extracted.

**Results:**

Out of 7427 records, ten studies were included. The estimated prevalence of loneliness was 38.6% (95% CI 3.7–73.5%, *I* ^2^ = 99.6, *P* < 0.001) among individuals with MCI. Moreover, the estimated prevalence of loneliness was 42.7% (95% CI 33.8–51.5%, *I*² = 90.4, *P* < 0.001) among individuals with dementia. The estimated prevalence of social isolation was 64.3% (95% CI 39.1–89.6%, *I*² = 99.6, *P* < 0.001) among individuals with cognitive impairment. Study quality was reasonably high. It has been found that living alone and more depressive symptoms are associated with a higher risk of loneliness among individuals with dementia.

**Conclusions:**

Social isolation, and in particular loneliness, are significant challenges for individuals with MCI and dementia. This knowledge can contribute to supporting successful ageing among such individuals. Future research in regions beyond Asia and Europe are clearly required. In addition, challenges such as chronic loneliness and chronic social isolation should be examined among individuals with MCI or dementia.

Individuals with mild cognitive impairment ((MCI) referring to the transitional state between normal ageing and dementia) and dementia (a progressive cognitive impairment syndrome mainly caused by Alzheimer’s disease) have to cope with many challenges. For instance, they often experience impairments in their functionality (e.g. difficulties with handling finances or preparing meals).^1^ Thus, they often demand extensive care and supervision.^2^ Because of their cognitive impairment, admission to nursing home is sometimes inevitable.

Such factors can markedly shape their social relationships and social activities. For example, having more unidirectional relationships, such as contact with professional carers, could change the quality of relationships. In this case, one-sided interactions where people living with dementia are cared for by professional carers may feel unsatisfactory. Family relationships can also change as a result of care.^3^ The increasing cognitive impairment could also be accompanied by a reduction in social activities (e.g. turning away from social engagement), eventually resulting in loneliness (perceived discrepancy between actual and desired social relationships, either in qualitative or quantitative terms^4^) and social isolation (lack of social activities^5^).

Loneliness and isolation are risk factors for poor health outcomes in later life.^6,7^ For example, they can not only increase the risk of mental disorders (e.g. depression or anxiety), but also the risk of cardiovascular diseases (e.g. coronary heart disease and stroke) and poor self-rated health.^8,9^ Previous research has also demonstrated a link between loneliness/isolation and poor sleep, as well as impaired cognitive functioning.^10,11^

For instance, a previous meta-analysis of longitudinal studies showed that loneliness was positively associated with an increased risk of MCI (odds ratio 1.14, 95% CI 1.05–1.23).^12^ Another meta-analysis showed that loneliness was also associated with an increased risk of dementia (relative risk 1.23, 95% CI 1.16–1.31).^13^ Comparably, low social isolation levels were associated with better cognitive functioning in late life (*r* = 0.05, 95% CI 0.04–0.06).^14^ In contrast to these frequently investigated associations between loneliness or social isolation and MCI or dementia, the prevalence of loneliness and social isolation among individuals with MCI or dementia has been much less researched.

However, there are some studies that examine the prevalence of loneliness/isolation among individuals with MCI or dementia. For example, Eshkoor et al^15^ found that 47.9% of community-dwelling individuals with dementia can be classified as socially isolated in Peninsular Malaysia. Other research^16^ has showed that 33.3% of community-dwelling individuals with MCI can be classified as lonely in Singapore. However, no systematic review/meta-analysis synthesising existing evidence has been undertaken. Therefore, our aim was to conduct the first systematic review and meta-analysis to investigate the prevalence and factors associated with loneliness and social isolation among individuals with MCI or dementia.

It is projected that the global number of individuals with dementia may increase from 57.5 million in 2019 (95% CI 50.4–65.1) to 152.8 million (95% CI 130.8–175.9) in 2050,^17^ a fact that underscores the relevance of our topic. Moreover, previous research has also suggested a steep increase in the number of individuals with MCI by 2060,^18^ further highlighting the relevance. This present work may determine potential antecedents and consequences of loneliness/social isolation among individuals with MCI or dementia. Furthermore, this work might shed light on present gaps in knowledge. This could further inspire future studies. Compared to single studies, meta-analyses can also yield a more accurate overview. Moreover, it is important because social isolation is a modifiable risk factor for dementia.^19^ Consequently, prevalence estimates are crucial for developing dementia prevention strategies. Furthermore, similar to older adults in general, one can assume that loneliness and social isolation predict subsequent poor physical and mental health outcomes among individuals with MCI or dementia. Thus, understanding the frequency of loneliness and social isolation is essential.

In terms of clinical implications, discussing and asking about loneliness and social isolation can improve patient–provider connections and health outcomes.^20^ For example, older adults are frequently worried about developing dementia.^21^ Thus, they might be highly motivated to tackle determinants such as isolation and loneliness.^22,23^

## Method

This current study satisfied the Preferred Reporting Items for Systematic Reviews and Meta-Analysis (PRISMA) guidelines.^24^ Our work was also registered in the International Prospective Register of Systematic Reviews (PROSPERO, registration number: CRD42024550504). Subsequent amendments were not made. Of note, we initially intended to perform a meta-regression. However, because of the number of studies included in meta-analysis, we refrained from doing a meta-regression. This procedure is in accordance with existing recommendations.^25^

In June 2024, an electronic search was done across five databases: PubMed, PsycINFO, CINAHL, Web of Science and Scopus. The search strategy for all electronic databases is shown in Supplementary File 1. Our search strategy (and the selection of databases) was also guided by a librarian’s advice, with whom we had intensive dialogue.

The relevance assessment was performed in two stages by two reviewers (A.H. and H.-H.K.): first, they screened the titles and abstracts independently, and then they examined the full texts independently, i.e. both steps were performed dually and independently. Additionally, a manual search was performed by reviewing the references of the included studies and checking for studies that cited those included. When there were differing opinions on study inclusion, discussions were held to reach a consensus.

The inclusion criteria of this work were as follows: (a) cross-sectional and longitudinal observational studies focusing on the prevalence of loneliness or social isolation among individuals with MCI and/or individuals with dementia, (b) use of appropriate tools for assessing key variables and (c) studies available in English or German and published in peer-reviewed scientific journals.

Grey literature was not examined. There were no restrictions regarding place and time of studies. An appropriate assessment for loneliness/isolation and MCI/dementia closely follows the criteria described in the Consensus-Based Standards for the Selection of Health Measurement Instruments (COSMIN) guidelines.^26^

A pre-test examining 100 titles/abstracts was first performed before determining the final inclusion criteria. However, our inclusion criteria were not altered. One author (A.H.) performed data extraction and a second author (H.-H.K.) checked the data extraction carefully. Data extraction (first extracted on 6 June 2024) covered the design of the study, measurement of loneliness/isolation, tool used to determine MCI/dementia, characteristics of the sample, analytical approach and main findings. When data were incomplete or unclear, the authors of the respective studies were contacted via email. We used Cohen’s kappa to evaluate the interrater agreement between the two authors (A.H., H.-H.K.). Cohen’s kappa was 0.83 for full-text selection.

The Joanna Briggs Institute standardised critical appraisal instrument, designed for prevalence studies, was used for assessing the quality of the studies.^27^ The final sum score varies from 0 to 9, whereby higher values reflect a better study quality and a lower risk of bias. The evaluation of the study quality was performed dually (A.H., H.-H.K.) and independently. Of note, we did not apply a specific cut-off for excluding studies from meta-analysis.

A random-effects model for meta-analysis was used because heterogeneity across studies was assumed. Following current recommendations, the *I*²-statistic was used to evaluate heterogeneity among studies (with *I*²-values 25–50% categorised as low, 50–75% as moderate and ≥75% as high heterogeneity).^28^

In our main analysis, we only distinguished between the presence and absence of loneliness. The absence of loneliness refers to ‘no, seldom’ or ‘no, never’ when replying to a single-item measure of loneliness. Moreover, it simply refers to ‘no’ when using a single item of loneliness distinguishing between the absence and presence of loneliness. Furthermore, established cut-offs were used that were applied in the studies included in this meta-analysis (see Table 1 for further details).

**Table 1:** Study overview and key findings

Author, year	Country	Assessment of loneliness/social isolation	Sample and study type	Time of data collection	Sample size, age in total sample and gender ratio	Results: prevalence of chronic loneliness (%)	Results: prevalence stratified by gender	Results: correlates and associated factors
Eshkoor et al, 2014^15^	Malaysia	Social isolation: LSNS-6 (cut-off: 12) Dementia: MMSE score less than 26	Determinants of Health Status among Older Malaysians’ study Community-dwelling adults Cross-sectional survey ≥60 years of age Peninsular Malaysia divided into four zones (North, South, West and Central)	Not reported	*N* = 1210 individuals with dementia Mean age: not reported Female: 63.8%	Socially isolated: 579/1210 (47.9%)	Not reported	Multiple logistic regressions showed that social isolation was significantly associated with higher odds of sleep disturbances (odds ratio 1.33, 95% CI 1.05–1.69)
Fang et al, 2020^40^	China	Social isolation: LSNS-6 (cut-off: 12) MCI: screening process involving seven steps (e.g. Clinical Dementia Rating above the cut-off for mild dementia; a consensus was made by psychiatrists, neurologists and neuropsychologists)	Multicentre, prospective cohort study Hospital-based: 12 hospitals (i.e. institutionalised) Longitudinal design ≥55 years of age, with MCI and *Helicobacter pylori*–infected peptic ulcer disease Guangdong, Fujian and Hunan provinces	January 2012 to November 2014 Follow-up: December 2014 to December 2017	*N* = 1900 individuals with MCI Mean age: 69.7 (s.d. 7.9) years Female: 48.8%	Socially isolated: 946/1900 (49.8%)	Not reported	Cox proportional hazard models showed that social isolation was significantly associated with a greater risk of peptic ulcer disease recurrence (hazard ratio 2.67, 95% CI 1.60–4.52) among individuals with MCI
Hajek et al, 2023^39^	Germany	Loneliness: Single item (1=never/almost never to 4 = always/almost always) Trichotomized: absence of loneliness (covering ‘never/almost never’), moderate loneliness (covering ‘sometimes’ and ‘mostly’) and severe loneliness (covering ‘always/almost always’) MCI/dementia: based on the DemTect (score ranges from 0 to 18, whereby higher values correspond to lower cognitive impairment) MCI: scores between 9 and 12 Dementia: scores lower than 9	Survey on quality of life and subjective well-being of the very old in North Rhine-Westphalia (NRW80+) Community-dwelling and institutionalised individuals Cross-sectional study Aged 80 years and over residing in North Rhine-Westphalia	August 2017 to February 2018	*n* = 199 individuals with MCI *n* = 116 individuals with dementia Mean age (among individuals with MCI): 86.9 (s.d. 4.6) years Mean age (among individuals with dementia): 88.3 (s.d. 4.1) years Female (among individuals with MCI): 45.2% Female (among individuals with dementia): 56.0%	Moderate loneliness (among individuals with MCI): 35/199 (17.6%) Severe loneliness (among individuals with MCI): 14/199 (7.0%) Moderate loneliness (among individuals with dementia): 30/116 (25.9%) Severe loneliness (among individuals with dementia): 15/116 (12.9%)	Women: moderate loneliness (among individuals with MCI): 19/90 (21.1%) Severe loneliness (among individuals with MCI): 6/90 (6.7%) Moderate loneliness (among individuals with dementia): 17/65 (26.2%) Severe loneliness (among individuals with dementia): 10/65 (15.4%) Men: Moderate loneliness (among individuals with MCI): 16/109 (14.7%) Severe loneliness (among individuals with MCI): 8/109 (7.3%) Moderate loneliness (among individuals with dementia): 13/51 (25.5%) Severe loneliness (among individuals with dementia): 5/51 (9.8%)	Not reported
Holmén et al, 2000^35^	Sweden	Loneliness (single item): ‘Do you often feel lonely?’ (no, yes) Dementia: MMSE: preliminary diagnostic procedure; final diagnosis of dementia by physicians following the DSM-III^58^	Kungsholmen project ‘Ageing and Dementia’ Community-dwelling and hospital/nursing home Longitudinal population-based survey Aged 75 years and over living in Stockholm	Not reported	*N* = 154 individuals with dementia Mean age: not reported specifically among individuals with dementia Female: 77.2%	Loneliness: 71/154 (46.1%)	Not reported	Not reported
Lampinen et al, 2022^36^	Sweden	Loneliness: single item (Do you ever feel lonely? yes, often; yes, sometimes; no, seldom; no, never). Dichotomisation: lonely (‘yes, often’, or ‘yes, sometimes’); Not lonely (‘no, seldom’ or ‘no, never’) Dementia: according to the DSM-III,^58^ verified based on information from medical records, prescriptions and assessments (e.g. MMSE)	Umeå 85 + /Gerontological Regional Database study Community-dwelling and institutionalised Population-based cohort study City of Umeå (85 years and over) and in five rural municipalities of Västerbotten County	2000/2002 (baseline) to 2017 (every 5 years)	*N* = 344 individuals with dementia Mean age (among lonely people): 90.8 (s.d. 5.0) years Mean age (among not lonely people): 90.4 (s.d. 4.4) years Female: 70.9%	Loneliness: 175/344 (50.9%)	Women: Loneliness: 137/244 (56.1%) Men: Loneliness: 38/100 (38.0%)	Multiple logistic regressions showed that living alone (odds ratio 6.65, 95% CI 2.26–19.55) and depressive symptoms (odds ratio 1.41, 95% CI 1.22–1.62) was significantly associated with a higher risk of loneliness, whereas other sociodemographic and social aspects, as well as aspects of social participation and medications and assessments, were not associated with loneliness among individuals with dementia
Nikmat et al, 2015^38^	Malaysia	Social isolation: Friendship Scale (0 to 24, higher scores reflect higher social connectedness) Cognitive impairment: according to the Short Mini-Mental State Examination (SMMSE) score with a cut-off of 10 out of 12 points^59^	Cross-sectional study Government nursing homes in West Malaysia Aged 60 years and over	Not reported	*N* = 110 with cognitive impairment Mean age: 71.6 (s.d. 7.8) years Female: 50%	Socially isolated: 16/110 (14.5%) Very socially isolated: 89/110 (80.9%)	Not reported	Not reported
Sung et al, 2024^16^	Singapore	Loneliness: UCLA 3-Item Loneliness Scale (sum score ranging from 3 to 9, with higher scores indicating higher loneliness levels). Dichotomised: absence of loneliness: 3; presence of loneliness: 4 or higher MCI: Eight-Item Informant Interview to Differentiate Aging and Dementia as well as two items from the MMSE	Caring for persons with dementia and their caregivers in the community: Towards a sustainable community based dementia care system (COGNITION) study Community-dwelling Cross-sectional design Aged 60 years and over residing in the Whampoa community	2018	*N* = 135 individuals with MCI (dyads, i.e. including 135 caregivers for the individuals with MCI) Mean age: 79.3 (s.d. 8.6) years Female: 51.9%	Loneliness: 44/135 (33.3%)	Not reported	Multiple logistic regressions showed that loneliness was not associated with loneliness of caregivers (*β* = 0.48, 95% CI –0.51 to 1.47).
Victor et al, 2020^33^	England, Scotland and Wales	Loneliness: Six-item De Jong Gierveld Loneliness Tool (score: 0–6; 0–1: no loneliness; 2–4: moderate loneliness; 5–6: severe loneliness) Dementia: clinical diagnosis of dementia (any subtype), MMSE of 15 or higher (i.e. mild to moderate dementia)	Improving the experience of Dementia and Enhancing Active Life (IDEAL) cohort Community-dwelling 29 National Health Service sites throughout England, Scotland and Wales Cross-sectional design	2014–2016 (baseline wave)	*N* = 1445 individuals with dementia Mean age: 76 (s.d. 8.6) years Female: 43.7%	Moderate loneliness: 435/1445 (30.1%) Severe loneliness: 75/1445 (5.2%)	Women: Moderate loneliness: 199/676 (29.4%) Severe loneliness: 37/676 (5.5%) Men: Moderate loneliness: 236/871 (27.1%) Severe loneliness: 38/871 (4.4%)	Multinomial regressions showed that depressive symptoms (e.g. relative risk 1.34, 95% CI 1.14–1.58, with severe loneliness as outcome) as well as higher social isolation levels (e.g. relative risk 0.86, 95% CI 0.81–0.91) were both associated with moderate/severe loneliness. Moreover, living alone (relative risk 5.01, 95% CI 1.65–15.28) and reporting poorer quality of life (relative risk 0.87, 95% CI 0.81–0.94) were associated with severe loneliness risk
Willmott et al, 2024^37^	England	Loneliness: Based on natural language processing (text-mining) MCI: Individuals receiving an MCI diagnosis according to ICD-10 criteria (F06.7)	Retrospective cohort study Aged 50 years and older Four South London boroughs	2007–2020	*N* = 2250 individuals with MCI Mean age: 75.0 (s.d. 10.8) years Female: 55.5%	Loneliness: 272/2250 (12.1%)	Not reported	Cox regressions showed that loneliness was significantly associated with dementia development (hazard ratio 1.56, 95% CI 1.12–2.16)
Zafar et al, 2021^34^	Pakistan	UCLA Loneliness Scale (20-item version): score ranges from 20 to 80, whereby higher scores reflect a high level of loneliness; 20–34: low degree of loneliness 35–49: moderate degree of loneliness 50–64: moderately high degree of loneliness; 65–80: high degree of loneliness MCI: MMSE was used to screen out MCI. Further information on cognitive function was collected by clinical interview and subjective assessment	Cross-sectional survey (purposive sampling) Old age homes in Lahore and Rawalpindi (*n* = 82); and community-dwelling in Sargodha (*n* = 88) Aged 60 years and over	Not reported	*N* = 170 individuals with MCI Mean age: 69.8 (s.d. 5.4) years Female: 50.0%	Low loneliness: 25/170 (14.7%) Moderate loneliness: 122/170 (71.8%) Moderately high loneliness: 23/170 (13.5%)	Not reported	Mediation analysis (according to Hayes^60^) showed that loneliness partially mediates (overall indirect effect, *b* = 0.046, 95% CI 0.002–0.156) the association between depression and quality of life among individuals with MCI

LSNS-6, Lubben Social Network Scale, 6-item version; MMSE, Mini-Mental State Examination; MCI, mild cognitive impairment; DemTect, Demenzdetektionstest; UCLA, University of California, Los Angeles.

It was initially intended to compute a funnel plot and perform the Egger test (with *P* < 0.05 indicating the presence of publication bias) to determine a possible publication bias.^29^ However, because of the small number of studies, we refrained from performing it. We used Stata version 18.0 for Windows (StataCorp, College Station, Texas, USA) for statistical analysis, i.e. meta-analysis. We also used the metaprop tool.^30^

## Results

### Study overview

A flowchart illustrating the search process is shown in Supplementary File 2.^24^ Overall, 7427 records were identified. First, duplicates were removed, resulting in 4217 hits, which were screened. The titles/abstracts were screened. The main reason for excluding studies was the lack of prevalence data on loneliness or social isolation among individuals with MCI or dementia. After this screening procedure, 48 full texts were examined in the next step. Most of these studies did not meet the inclusion criteria, frequently because they did not provide prevalence data for the groups of interest. Some studies were also excluded from the full-text screening because they described loneliness among individuals who only later developed MCI/dementia.^31,32^ In this systematic review and meta-analysis, ten studies were included in total (three of these studies were identified in the manual hand search).^15,16,33–40^

Main characteristics and key findings of the studies are provided in Table 1. Adjusted findings are presented in Table 1. The studies were exclusively from Europe and Asia: two studies from Sweden, two studies from England (thereof, one study included data from England/Scotland/Wales), two studies from Malaysia and one study each from Germany, China, Pakistan and Singapore. Six studies had a cross-sectional design, and four studies had a longitudinal design. Five studies included both community-dwelling and institutionalised individuals, whereas three other studies exclusively included community-dwelling individuals, and two studies exclusively included individuals residing in institutionalised settings such as nursing homes. The studies were partly based on well-known samples such as the Improving the experience of Dementia and Enhancing Active Life (IDEAL) cohort or the Umeå 85+/Gerontological Regional Database study, whereas other studies conducted surveys/examinations on their own. The established samples in particular had large cohorts of about 1000–2000 individuals with MCI or dementia, whereas the remaining studies included about 100–400 individuals with MCI or dementia. All studies included both women and men, with the proportion of women mainly ranging from approximately 40 to 70%. The average age of study participants often ranged from about 70 to 90 years across the studies. Single-item tools were most frequently used to measure loneliness, followed by University of California, Los Angeles (UCLA) tools^41^ and the De Jong Gierveld (DJG) tool.^42,43^ Two studies used the Lubben Social Network Scale, 6-item version (LSNS-6) tool^44^ and one study used the friendship scale to quantify social isolation. Established screening tools (e.g. Mini-Mental State Examination^45^) were mainly used to measure MCI or dementia.

The publication dates ranged from 2000 to 2024, with seven studies published in 2020 or later, and the remaining three studies published in 2000, 2014 and 2015, respectively. The date of data collection is not clear for all studies, but based on the publication date, it can be assumed that all studies used data from before the COVID-19 pandemic. Additional details are shown in Table 1.

In total, four studies reported the prevalence of loneliness among individuals with MCI and four studies reported the prevalence of loneliness among individuals with dementia (one study reported both the prevalence of loneliness among individuals with MCI and individuals with dementia^39^). In addition, one study reported the prevalence of social isolation among individuals with MCI, one study reported the prevalence of social isolation among individuals with dementia and one further study reported the prevalence of social isolation among individuals with cognitive impairment (without further distinguishing between MCI and dementia).

### Meta-analysis

**Fig. 1:**
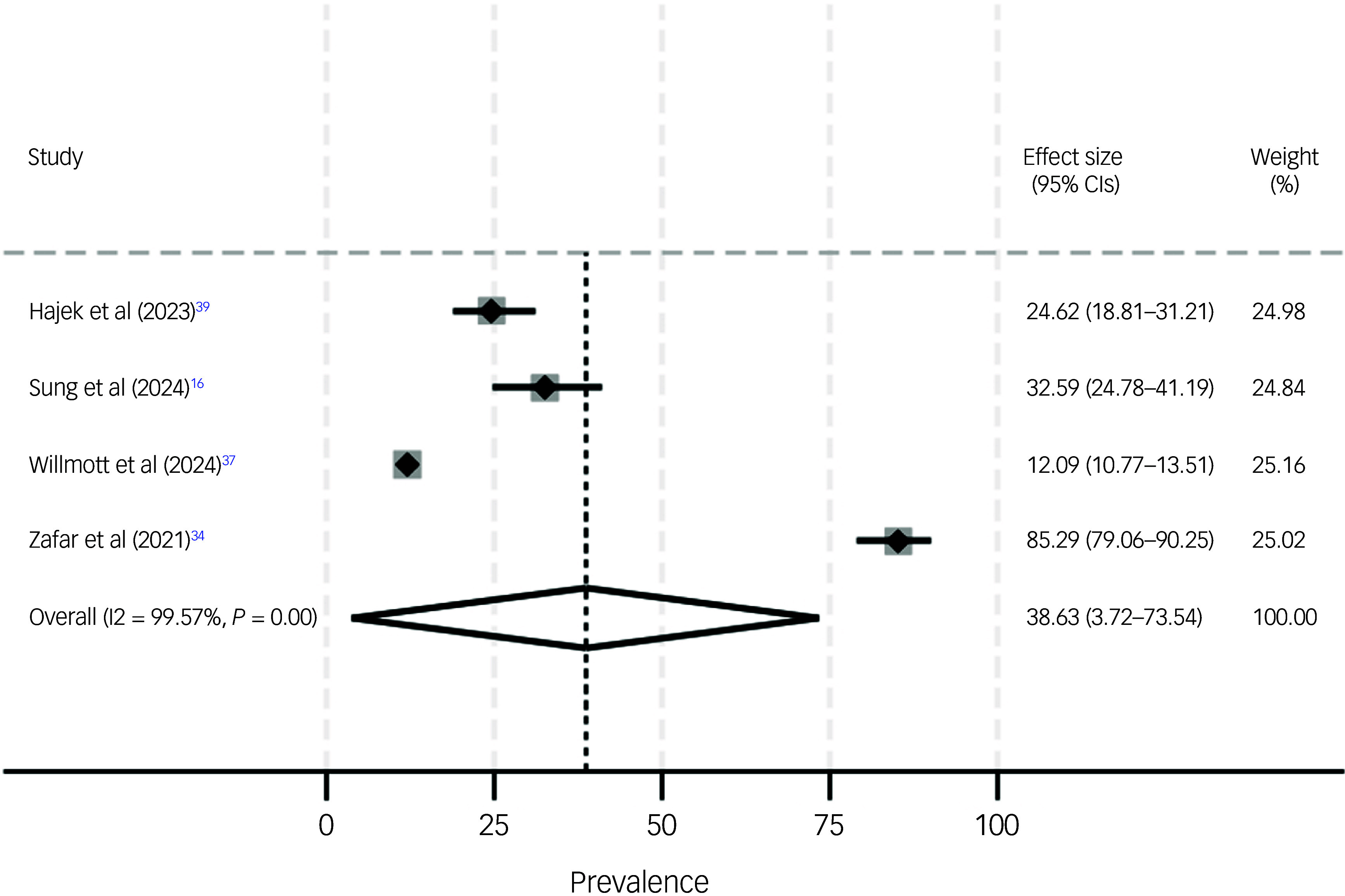
Meta-analysis of loneliness among individuals with mild cognitive impairment.

The estimated prevalence of loneliness was 38.6% (95% CI 3.7–73.5%, *I*² = 99.6, *P* < 0.001) among individuals with MCI (see Fig. 1). When the study conducted by Zafar et al^34^ (which had a very high prevalence of loneliness) was excluded from meta-analysis, the estimated prevalence of loneliness decreased to 22.7% (95% CI 9.9–35.4%, *I*² = 95.0, *P* < 0.001) among individuals with MCI.

**Fig. 2:**
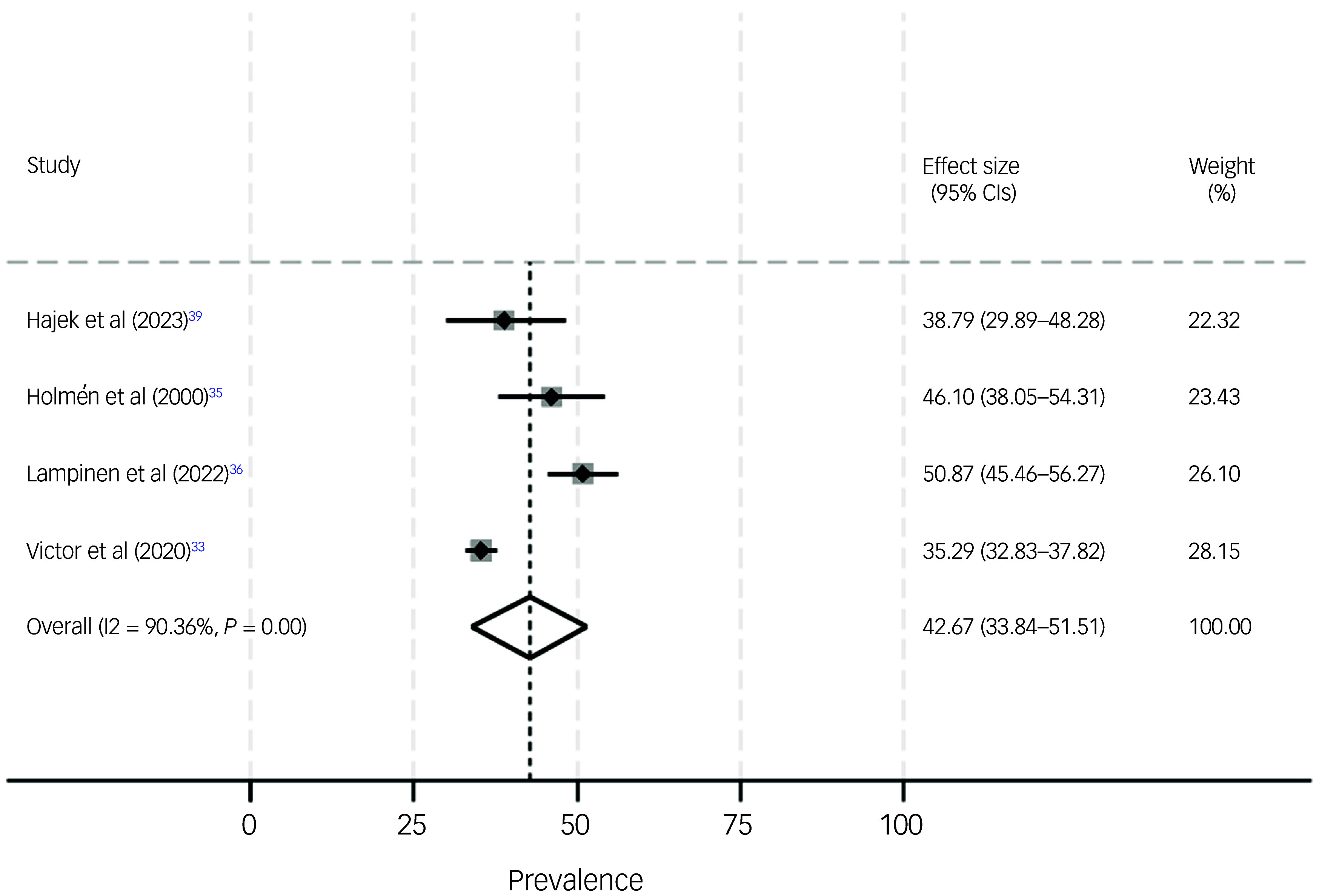
Meta-analysis of loneliness among individuals with dementia.

In addition, the estimated prevalence of loneliness was 42.7% (95% CI 33.8–51.5%, *I*² = 90.4, *P* < 0.001) among individuals with dementia (see Fig. 2). Of note, stratified by gender, the estimated prevalence of loneliness was 44.2% (95% CI 28.9–59.5, *I*² = 94.0, *P* < 0.01) among women with dementia and 32.2% (95% CI 29.4–35.1%, *I*² = 0.0, *P* = 0.39) among men with dementia. A meta-analysis for loneliness stratified by gender for individuals with MCI was not possible because of the lack of studies. We also conducted a meta-analysis with prevalence of loneliness for other subgroups among individuals with MCI (Table 2) and individuals with dementia (Table 3).

**Table 2:** Subgroup analysis of the pooled prevalence of loneliness (among individuals with mild cognitive impairment)

Characteristics	Subgroups	Number of studies	Prevalence	95% CI	*I*² (%), *P*-value
Region	Europe	2	12.7	11.4–14.0	
	Asia	2	68.9	64.4–73.3	
Tools used to quantify loneliness	Multi-item scales/text mining^a^	3	43.3	0–92.7	99.7, *P* < 0.01
Living arrangement	Community-dwelling and institutionalised^b^	3	40.6	0–85.1	99.7, *P* < 0.01
Quality assessment score	Score of 8 or higher	2	12.7	11.4–14.0	
	Score of 7 or lower	2	68.9	64.4–73.3	

a. Because of the lack of studies, a meta-analysis could not be conducted with studies only including single-item tools.

b. Because of the lack of studies, a meta-analysis could not be conducted with studies only including community-dwelling individuals or institutionalised individuals.

**Table 3:** Subgroup analysis of the pooled prevalence of loneliness (among individuals with dementia)

Characteristics	Subgroups	Number of studies	Prevalence	95% CI	*I*² (%), *P*-value
Region	Europe^a^	4	42.7	33.8–51.5	90.4, *P* < 0.01
Tools used to quantify loneliness	Single item^b^	3	46.0	39.1–52.8	62.8, *P* = 0.07
Living arrangement	Community-dwelling and institutionalised^c^	3	46.0	39.1–52.8	62.8, *P* = 0.07
Quality assessment score	Score of 8 or higher^d^	3	41.6	30.7–52.5	92.7, *P* < 0.01

a. Because of the lack of studies, a meta-analysis could not be conducted with studies only including individuals from Asia.

b. Because of the lack of studies, a meta-analysis could not be conducted with studies only including multi-item tools.

c. Because of the lack of studies, a meta-analysis could not be conducted with studies only including community-dwelling individuals or institutionalised individuals.

d. Because of the lack of studies, a meta-analysis could not be conducted with studies having a quality score of 7 or lower.

With respect to severity of loneliness experienced by the sample (i.e. moderate and severe loneliness), the estimated prevalence of moderate and severe loneliness was 38.1% (95% CI 34.0–42.3%, *I*² = 0.0) and 9.1% (95% CI 6.2–12.1%, *I*² = 0.0), respectively, among individuals with MCI. The estimated prevalence of moderate and severe loneliness was 29.8% (95% CI 27.5–32.0%, *I*² = 0.0) and 5.5% (95% CI 4.3–6.6%, *I*² = 0.0), respectively, among individuals with dementia.

**Fig. 3:**
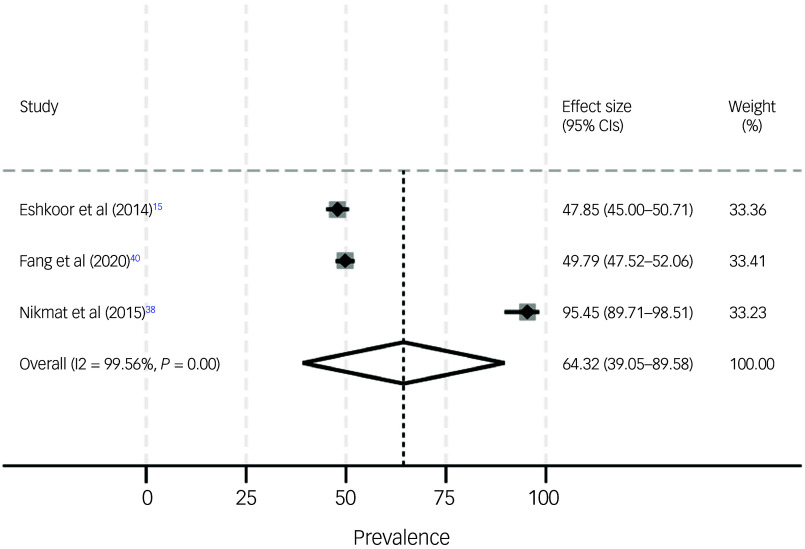
Meta-analysis of social isolation among individuals with cognitive impairment.

One study examined social isolation among individuals with dementia, a second study examined social isolation among individuals with MCI and a third study investigating social isolation did not differentiate between MCI and dementia. We decided to consider all three studies in the meta-analysis, to provide a first impression of the prevalence of social isolation among individuals with cognitive impairment; the estimated prevalence of social isolation was 64.3% (95% CI 39.1–89.6%, *I*² = 99.6, *P* < 0.001) among individuals with cognitive impairment (see Fig. 3). The prevalence of social isolation reduced to 49.0% (95% CI 47.3–50.8%) among individuals with cognitive impairment when the study by Nikmat et al^38^ (which had an extraordinarily high prevalence of social isolation) was removed from the meta-analysis.

### Loneliness: predictors and outcomes

Two studies investigated the predictors of loneliness among individuals with dementia. Both studies showed that living alone and depressive symptoms were associated with a higher loneliness risk in some European countries.^33,36^

Two studies examined the outcomes of loneliness among individuals with MCI. One study showed that loneliness can contribute to the development of dementia in such group.^37^ Another study showed that loneliness of individuals with MCI (care recipients) was not associated with loneliness of caregivers, based on dyadic data.^16^

A further study showed that loneliness partially mediates the link between depression and quality of life among individuals with MCI.^34^

### Social isolation: predictors and outcomes

Two studies examined the consequences of social isolation among individuals with MCI or dementia. One study showed that social isolation was significantly associated with higher odds of sleep disturbances among individuals with dementia.^15^ A second study showed that social isolation was significantly associated with a greater risk of peptic ulcer disease recurrence among individuals with MCI.^40^ None of the studies examined the determinants of social isolation among individuals with MCI or dementia.

### Quality assessment/risk-of-bias assessment

The assessment of the study quality/risk of bias is shown in Supplementary File 3. The scores varied from 5 to 9 (mean 7.2, s.d. = 0.8), corresponding to a quite good level in total, with a quite low bias risk. The unclear or insufficient presentation or management of low response rates was the most common limitation observed across included studies.

## Discussion

Our systematic review and meta-analysis showed that loneliness and social isolation are highly prevalent in people with dementia and MCI. This finding has important implications for prevention strategies aimed at reducing the disease burden of these conditions.

A previous meta-analysis revealed a pooled prevalence of loneliness of 28.6% (95% CI 22.9–35.0%) among individuals aged 65 years and over during the COVID-19 pandemic, covering 15 countries across Asia, Europe, North America and South America.^46^ This work also revealed a pooled prevalence of social isolation of 31.2% (95% CI 20.2–44.9%). Not surprisingly, we found markedly higher prevalence rates in our current work. In our view, this can be explained primarily by the differences in the cognitive impairments of the populations studied.

Comparing the prevalence of loneliness among individuals with MCI and dementia, the rates seem to differ only moderately. However, when the study^34^ with a very high prevalence of loneliness in individuals with MCI was omitted from the meta-analysis, there were much more pronounced differences in loneliness among individuals with MCI compared with individuals with dementia. These greater differences would align with former meta-analyses identifying a weaker association between loneliness and risk of MCI^12^ compared with the association between loneliness and dementia.^13^ Of note, our meta-analyses for corresponding subgroups (e.g. stratified by continent) must be interpreted with great caution because of the small number of studies. We would therefore like to refrain from discussing these in depth in this current work.

Two included studies determined that living alone and more depressive symptoms were associated with a higher loneliness risk among individuals with dementia.^33,36^ Very similar results have been identified by a former systematic review/meta-analysis investigating the prevalence and correlates of loneliness/social isolation among the most eldery.^47^ Because of the paucity of literature on the predictors and outcomes of loneliness/isolation in people with MCI or dementia, no further reliable conclusions can be drawn. Rather, this lack of studies stresses the need for further research.

The mean quality of the included studies was quite high. However, several studies did not clarify the response rate or clarify how they managed low response rates. Given the fact that older individuals with cognitive impairment were examined, it is reasonable that the (not reported) response rates in the studies might actually be rather low. It is also plausible that individuals with more severe cognitive impairments had a lower participation rate, suggesting a potential sample selection bias. In this respect, the generalisability of the samples may not always be fully given. This should be acknowledged as a potential limitation of the studies included in this work.

Some knowledge gaps were identified. Overall, there are only a few studies on this topic. For instance, there is a clear need for future studies examining social isolation among individuals with MCI and dementia. Although loneliness can be measured with single-item tools,^48^ upcoming research with multi-item tools, such as the DJG tool^43^ or the ALONE scale (a tool specifically developed for older adults) by Deol et al,^49^ would be desirable to better capture the complexity of loneliness. Moreover, an additional external assessment of loneliness may also be useful because it may be the case that self-ratings differ from external ratings because of, among other things, language barriers of individuals with MCI or dementia. Future research in this area is recommended. Furthermore, the types of loneliness (emotional versus social loneliness) and subtypes of dementia (e.g. vascular dementia or Alzheimer’s disease) could be examined in future research. Furthermore, long-running studies (with large samples) would be desirable to better identify the factors leading to loneliness or social isolation – in particular, chronic states of loneliness and social isolation – and their consequences among individuals with MCI or dementia (e.g. based on the Social Relationship Expectations Framework).^50^ Moreover, a greater geographical diversity when examining loneliness/isolation among individuals with MCI or dementia is clearly required. Thus, we encourage research from North America, South America, Oceania and Africa. Individuals with MCI or dementia had a particularly difficult time during the COVID-19 pandemic (e.g. because of contact restrictions).^51^ We would therefore also recommend future research during and after the pandemic. Although it should be acknowledged that some studies have already included individuals living in institutionalised settings, we would like to stress the need for further research in this setting (which is often associated with more severe cognitive impairment^52^).

It is important to acknowledge the strengths and limitations of our present systematic review and meta-analysis. This study is the first systematic review and meta-analysis specifically investigating the prevalence of loneliness and social isolation among individuals with MCI or dementia. Important procedures were performed independently by two reviewers. Our work adheres to existing guidelines and was preregistered (PROSPERO). We also conducted a meta-analysis. A notable limitation is our restriction to peer-reviewed articles, which may have led to the exclusion of relevant studies. However, this approach was chosen to maintain a high standard of study quality. Five comprehensive databases were used, although this choice might have still resulted in the exclusion of appropriate studies. However, we assume that we were able to find most of the key studies by using these large databases in combination with the additional hand search. Because of the number of studies, we did not perform a meta-regression, in accordance with existing recommendations.^25^ However, if sufficient studies were available in the future, we would encourage future work to perform meta-regressions to uncover possible differences, e.g. in ethnicity, living situation, coping resources or personality.

The high prevalence of loneliness and social isolation among individuals with dementia or MCI indicates a need for public health strategies aimed at alleviating the disease burden caused by loneliness. Implementing such strategies could potentially reduce the incidence of dementia and MCI, and may also enhance outcomes for those already diagnosed with dementia or MCI. Of note, Borjali and Taheri^53^ recently proposed a multifaceted approach including public awareness campaigns, community-based interventions and training for healthcare providers. Moreover, based on an umbrella review of former systematic reviews and meta-analyses, Veronese et al concluded that meditation/mindfulness, social cognition training and social support interventions can reduce loneliness.^54^

Perissinotto et al also described individual clinical ways to tackle loneliness. These ways may include improving social skills, enhancing social support, finding opportunities for social interactions and tackling maladaptive social cognition^20^ (see also Masi et al^55^). For example, improving social skills may involve psychotherapy for people who have problems with social interactions or relationships.^20^ To improve social support, health professionals need to identify what is missing in a person’s life and use available community resources.^20^ Opportunities for social interactions can be improved by a variety of factors. One simple way may be hearing aids.^20^ Hearing impairments are also quite common among older adults with MCI or dementia.^56^ Other efforts could focus on strengthening transportation when being functionally impaired,^20^ which often co-occurs with MCI and dementia.^57^ Additionally, cognitive–behavioural therapy may support individuals in reframing harmful beliefs that affect their social interactions, which may require the involvement of behavioural health specialists to support emotional coping with critical life events that may lead to loneliness.^20^ Other research has also suggested including screening tools for isolation and loneliness in electronic health records.^20^

In conclusion, social isolation, and particularly loneliness, are significant challenges for individuals with MCI or dementia. Knowledge summarised by this study can help to improve the quality of life of individuals with MCI or dementia. Future research in regions beyond Asia and Europe are clearly required. Additionally, challenges such as chronic loneliness and chronic social isolation should be examined among individuals with MCI or dementia.

## Supporting information

Hajek and König supplementary material 1 Hajek and König supplementary material

Hajek and König supplementary material 2 Hajek and König supplementary material

Hajek and König supplementary material 3 Hajek and König supplementary material

## Data Availability

Data availability is not applicable to this article as no new data were created.

## Supplementary material

The supplementary material is available online at https://doi.org/10.1192/bjo.2024.865
