# Supplementary material for: Prevalence of loneliness and social isolation among individuals with mild cognitive impairment or dementia: systematic review and meta-analysis
Source: BJPsych Open. 2025 Mar 11;11(2):e44. doi: 10.1192/bjo.2024.865 (PMC12001938; doi:10.1192/bjo.2024.865)
Supplement: Hajek and König supplementary material 1 — Hajek and König supplementary material [file S2056472424008652sup001.docx]

Additional File 1. Search strategy (PubMed, CINAHL, PsycInfo, Web of Science and Scopus)

PubMed search algorithm

| Number | Search term |
| --- | --- |
| #1 | Social isolation [MeSH Terms] |
| #2 | Social exclu* |
| #3 | Lonel* |
| #4 | #1 OR #2 OR #3 |
| #5 | Dementia [MeSH Terms] |
| #6 | Mild cognitive impairment |
| #7 | #5 OR #6 |
| #8 | #4 AND #7 |

CINAHL search algorithm:

| S1 | “Social isolat*” |
| --- | --- |
| S2 | “Social exclu*” |
| S3 | “Lonel*” |
| S4 | S1 OR S2 OR S3 |
| S5 | Dementia |
| S6 | Mild cognitive impairment |
| S7 | S5 OR S6 |
| S8 | S4 AND S7 |

PsycInfo search algorithm:

| 1 | Social isolat*.ti |
| --- | --- |
| 2 | Social isolat*.ab |
| 3 | Social exclu*.ti |
| 4 | Social exclu*.ab |
| 5 | Lonel*.ti |
| 6 | Lonel*.ab |
| 7 | 1 OR 2 OR 3 OR 4 OR 5 OR 6 |
| 8 | Dementia.ti |
| 9 | Dementia.ab |
| 10 | Mild cognitive impairment.ti |
| 11 | Mild cognitive impairment.ab |
| 12 | 8 OR 9 OR 10 OR 11 |
| 13 | 7 AND 12 |

Web of Science search algorithm:

| 1 | TS=(Social isolat*) |
| --- | --- |
| 2 | TS =(Social exclu*) |
| 3 | TS=(Lonel*) |
| 4 | #1 OR #2 OR #3 |
| 5 | TS=(Dementia) |
| 6 | TS=(Mild cognitive impairment) |
| 7 | #5 OR #6 |
| 8 | #4 AND #7 |

Scopus search algorithm:

| 1 | TITLE-ABS-KEY ( {social isolation} ) |
| --- | --- |
| 2 | TITLE-ABS-KEY ( {social exclusion} ) |
| 3 | TITLE-ABS-KEY ( {loneliness} ) |
| 4 | 1 OR 2 OR 3 |
| 5 | TITLE-ABS-KEY ( {dementia} ) |
| 6 | TITLE-ABS-KEY ( {mild cognitive impairment} ) |
| 7 | 5 OR 6 |
| 8 | 4 AND 7 |
