# Supplementary material for: Prevalence of loneliness and social isolation among individuals with mild cognitive impairment or dementia: systematic review and meta-analysis
Source: BJPsych Open. 2025 Mar 11;11(2):e44. doi: 10.1192/bjo.2024.865 (PMC12001938; doi:10.1192/bjo.2024.865)
Supplement: Hajek and König supplementary material 2 — Hajek and König supplementary material [file S2056472424008652sup002.docx]

**Identification of studies via databases and registers**

Records removed before screening:

Duplicate records removed (n = 3,210)

Records identified from*:

Databases (n = 7,427)

(Pubmed, n = 1,631)

(PsycINFO, n = 657)

(CINAHL, n = 1,045)

(Web of Science = 2,729)

(Scopus = 1,365)

**Identification**

Records screened

(n = 4,217)

Records excluded**

(n = 4,169)

Reports sought for retrieval

(n = 48)

Reports not retrieved

(n = 0)

**Screening**

Reports excluded: n=38

Prevalence not reported for loneliness/social isolation among individuals with MCI/dementia (n=25)

No peer-reviewed article (e.g., conference presentation) (n=12)

Qualitative study (n=1)

Reports assessed for eligibility

(n = 48)

Studies included in review

(n =10, thereof n=3 included via hand search)

All n=10 studies were included in meta-analysis

**Included**

*From:*  Page MJ, McKenzie JE, Bossuyt PM, Boutron I, Hoffmann TC, Mulrow CD, et al. The PRISMA 2020 statement: an updated guideline for reporting systematic reviews. BMJ 2021;372:n71. doi: 10.1136/bmj.n71

For more information, visit: <http://www.prisma-statement.org/>
