# Supplementary material for: Prevalence of loneliness and social isolation among individuals with mild cognitive impairment or dementia: systematic review and meta-analysis
Source: BJPsych Open. 2025 Mar 11;11(2):e44. doi: 10.1192/bjo.2024.865 (PMC12001938; doi:10.1192/bjo.2024.865)
Supplement: Hajek and König supplementary material 3 — Hajek and König supplementary material [file S2056472424008652sup003.docx]

Additional File 3. Quality assessment/risk of bias assessment

|  | Items | | | | | | | | | Quality score (from 1 to 9; higher scores indicate less risk of bias) |
| --- | --- | --- | --- | --- | --- | --- | --- | --- | --- | --- |
| Study | 1 | 2 | 3 | 4 | 5 | 6 | 7 | 8 | 9 | Total |
| Eshkoor et al. 2014 | Y | Y | Y | Y | Y | Y | Y | Y | U | 8 |
| Fang et al., 2020 | Y | Y | Y | Y | Y | Y | Y | Y | Y | 9 |
| Hajek et al., 2023 | Y | Y | Y | Y | Y | Y | Y | Y | N | 8 |
| Holmen et al., 2000 | Y | Y | N | N | Y | Y | Y | N | U | 5 |
| Lampinen et al., 2022 | Y | Y | Y | Y | Y | Y | Y | Y | U | 8 |
| Nikmat et al., 2015 | Y | N | N | Y | Y | Y | Y | N | U | 5 |
| Sung et al., 2024 | Y | Y | N | Y | Y | Y | Y | Y | U | 7 |
| Victor et al., 2020 | Y | Y | Y | Y | Y | Y | Y | Y | N | 8 |
| Willmott et al., 2024 | Y | Y | Y | Y | Y | Y | Y | Y | U | 8 |
| Zafar et al., 2021 | Y | N | N | Y | Y | Y | Y | Y | U | 6 |

Notes: The Joanna Briggs Institute (JBI) standardized critical appraisal tool for prevalence studies was used. The criteria comprised the following aspects, each receiving a response of yes (Y), no (N), or unclear (U): 1 = Suitability of the sample frame; 2 = Suitability of participant selection; 3 = Sufficiency of sample size; 4 = Characterization of study participants and setting; 5 = Data analysis: appropriate coverage of the identified sample; 6 = Employment of valid methods for condition identification; 7 = Standard/reliable evaluation of the condition; 8 = Suitability of the analytical method; 9 = Sufficiency of the response rate or appropriate handling of a low response rate, if needed.
